# Integrated small RNA, mRNA and protein omics reveal a miRNA network orchestrating metabolic maturation of the developing human heart

**DOI:** 10.1186/s12864-023-09801-8

**Published:** 2023-11-23

**Authors:** Adar Aharon-Yariv, Yaxu Wang, Abdalla Ahmed, Paul Delgado-Olguín

**Affiliations:** 1 Translational Medicine, The Hospital for Sick Children, 686 Bay Street, Toronto, Ontario M5G0A4 Canada; 2 Department of Molecular Genetics, Temerty Faculty of Medicine, University of Toronto, Toronto, Ontario Canada; 3 Heart & Stroke, Richard Lewar Centre of Excellence, Toronto, Ontario Canada

**Keywords:** Human fetal heart development, Small non-coding RNA, microRNA, Transcriptomics, Proteomics, Cardiac maturation, Cell cycle progression, Metabolic maturation, Energy homeostasis, Transcriptional regulatory networks

## Abstract

**Background:**

As the fetal heart develops, cardiomyocyte proliferation potential decreases while fatty acid oxidative capacity increases in a highly regulated transition known as cardiac maturation. Small noncoding RNAs, such as microRNAs (miRNAs), contribute to the establishment and control of tissue-specific transcriptional programs. However, small RNA expression dynamics and genome-wide miRNA regulatory networks controlling maturation of the human fetal heart remain poorly understood.

**Results:**

Transcriptome profiling of small RNAs revealed the temporal expression patterns of miRNA, piRNA, circRNA, snoRNA, snRNA and tRNA in the developing human heart between 8 and 19 weeks of gestation. Our analysis demonstrated that miRNAs were the most dynamically expressed small RNA species throughout mid-gestation. Cross-referencing differentially expressed miRNAs and mRNAs predicted 6200 mRNA targets, 2134 of which were upregulated and 4066 downregulated as gestation progressed. Moreover, we found that downregulated targets of upregulated miRNAs, including *hsa-let-7b*, *miR-1-3p*, *miR-133a-3p*, *miR-143-3p*, *miR-499a-5p*, and *miR-30a-5p* predominantly control cell cycle progression. In contrast, upregulated targets of downregulated miRNAs, including *hsa-miR-1276*, *miR-183-5p*, *miR-1229-3p*, *miR-615-3p*, *miR-421*, *miR-200b-3p* and *miR-18a-3p*, are linked to energy sensing and oxidative metabolism. Furthermore, integrating miRNA and mRNA profiles with proteomes and reporter metabolites revealed that proteins encoded in mRNA targets and their associated metabolites mediate fatty acid oxidation and are enriched as the heart develops.

**Conclusions:**

This study presents the first comprehensive analysis of the small RNAome of the maturing human fetal heart. Our findings suggest that coordinated activation and repression of miRNA expression throughout mid-gestation is essential to establish a dynamic miRNA-mRNA-protein network that decreases cardiomyocyte proliferation potential while increasing the oxidative capacity of the maturing human fetal heart. Our results provide novel insights into the molecular control of metabolic maturation of the human fetal heart.

**Supplementary Information:**

The online version contains supplementary material available at 10.1186/s12864-023-09801-8.

## Background

The heart is one of the first functional organs to develop during mammalian embryogenesis [1]. Complex cellular and molecular interactions coordinate the development of multiple cell lineages that form the four-chambered heart by the end of human gestational week seven [2, 3]. As gestation proceeds, the fetal heart adapts structurally and physiologically to sustain fetal life in the hypoxemic intrauterine environment [3–5]. By the eighth week of gestation, the heart begins to mature, the myocardium compacts and increases its contractile function, aligning with the growing fetus’ heart workload [6–9]. Metabolism in the fetal heart relies primarily on glucose as a major energy source for proliferating cardiomyocytes [10, 11]. However, as the heart continues to develop and cardiomyocytes differentiate and mature, genes involved in fatty acid metabolism and oxidative stress are upregulated [12, 13]. This leads to a significant increase in the mitochondrial oxidative capacity of the heart. Parallel to such a metabolic shift, the proliferation potential of differentiated cardiomyocytes decreases [10–13]. Previous investigation of the dynamic mRNA profile of the developing human heart revealed that metabolism-related and cell cycle regulation functions are the most enriched amongst genes that increase and decrease in expression as gestation progresses, respectively [14]. However, the fetal gene regulatory networks controlling cardiac maturation and metabolism are yet to be fully understood.

In recent years, a growing interest has emerged on small non-coding RNAs (sncRNAs) as regulators of development and many other biological processes [15–18]. Non-coding RNA species include transfer RNA (tRNA), ribosomal RNA (rRNA), small nuclear RNA (snRNA), small nucleolar RNA (snoRNA), microRNA (miRNA), piwi-interacting RNA (piRNA) and circular RNA (circRNA) [16]. Most sncRNAs function as part of RNA-protein complexes (RNP), primarily acting through RNA interference (miRNA, piRNA), RNA modification (snoRNA), and spliceosomal control (circRNA, snRNA) [17, 19]. Interestingly, sncRNAs are often expressed in a spatial and temporal-specific manner, with highly dynamic expression in specific cellular, environmental, and developmental contexts [17]. Although certain small RNA species, primarily miRNAs and circRNAs, regulate cardiomyocyte fate [20–26], most small RNA populations have not been comprehensively characterized in the human heart.

miRNAs target messenger RNA molecules, leading to their degradation, decay, or translational inhibition [27, 28]. miRNAs are transcribed in immature RNAs known as primary miRNAs (pri-miRNAs), which form a stem-loop secondary structure that is cleaved by various endonucleases to produce a mature miRNA [29]. Mature miRNAs bind the 3’ untranslated region (UTR) of target mRNAs in a sequence-specific manner via Argonaute-proteins [30, 31]. The relevance of miRNAs in the mammalian heart is underscored by the effect of global knockout of Dicer, a critical endonuclease required for miRNA maturation [21, 32–34]. Loss-of-function of Dicer in the embryonic mouse heart results in dilated cardiomyopathy and lethality due to heart failure [32, 34]. miRNAs specific to skeletal and cardiac muscle [21, 35, 36] (myomiRs), include *miR-1, miR-133, miR-143, miR-206* and *miR-208*, while *miR-1, miR-133* and *miR-208b* are enriched in the developing heart [37–39]. Non-myomiRs are also involved in cardiac development and regeneration. For example, *miR-130a*, which is enriched in the heart, kidney, liver, testis, and lung, regulates myocardium growth [40, 41]. Other miRNAs: *miR-17-92, miR-15*, and *miR-499*, regulate proliferation and differentiation of cardiac progenitors and cardiomyocytes in the developing heart [21, 42–44]. Nevertheless, the function of only a few miRNAs in fetal heart development has been investigated, and the full extent of their regulatory networks is yet to be established [40, 45, 46].

In this study, we reveal the expression profile of small RNA species in the human fetal heart. Furthermore, we integrated miRNA, mRNA and protein omics to uncover a regulatory network controlling metabolic maturation of the human fetal heart during mid-gestation. By identifying candidate miRNAs with potential functions in normal cardiac development, our research advances the current understanding of fetal heart development and its regulatory mechanisms.

## Results

### Transcriptome-wide sequencing of small RNA species in the developing human fetal heart

The transcriptional landscape of small RNA species in the developing human heart has yet to be comprehensively analyzed. To characterize the expression profile of small RNAs in the fetal heart as it develops, we sequenced small RNA libraries from 37 human fetal hearts between 8 and 19 weeks of gestation (Additional file 1). This time window encompasses a crucial phase of human fetal development characterized by substantial organ growth [47].

**Fig. 1:**
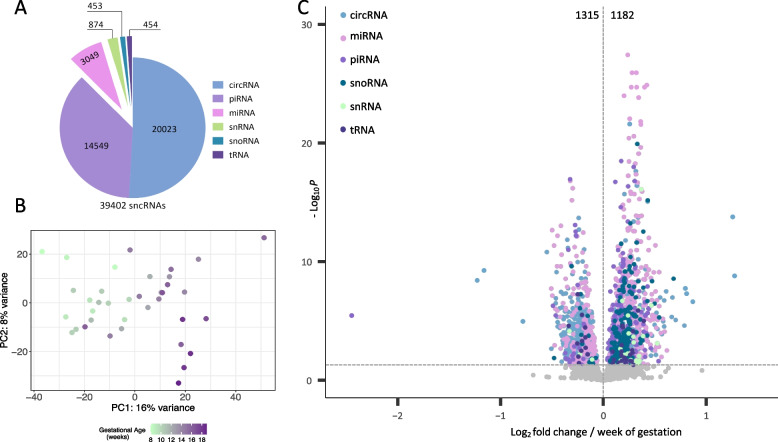
Dynamic expression of small RNA species in the developing human fetal heart. **A** Proportions of six small RNA subtypes making up the small RNAome of the human fetal heart. **B** Principal component analysis (PCA) plot separating cardiac small RNA expression profiles by gestational age. **C** Volcano plot of all the detected small RNAs. Differentially expressed small RNAs are in blue (circRNA), pink (miRNA), light purple (piRNA), dark blue (snoRNA), green (snRNA) and dark purple (tRNA) (*P* < 0.05). Abbreviations: sncRNA, Small noncoding RNA; circRNA, Circular RNA; miRNA, microRNA; piRNA, Piwi-interacting RNA; snoRNA, Small nucleolar RNA; snRNA, Small nuclear RNA; tRNA, Transfer RNA

Across all samples, the predominant small RNA species were circRNAs, accounting for 51% of the small noncoding RNAome. piRNAs constituted 37%, miRNAs 8%, while snRNAs, snoRNA, and tRNA collectively constituted 4% of the small RNA population (Fig. 1A). Small RNA levels separated fetal heart samples by gestational age on PC1, which explained 16% of variation (Fig. 1B). A total of 2497 small RNAs were differentially expressed across gestational age (*P* value < 0.05), of which 1315 (53%) were downregulated, and 1182 (47%) upregulated (Fig. 1C, Additional file 2, Additional file 3: Fig. S1A).

### miRNAs are the most dynamically expressed subtype of small RNAs in the fetal heart

**Fig. 2:**
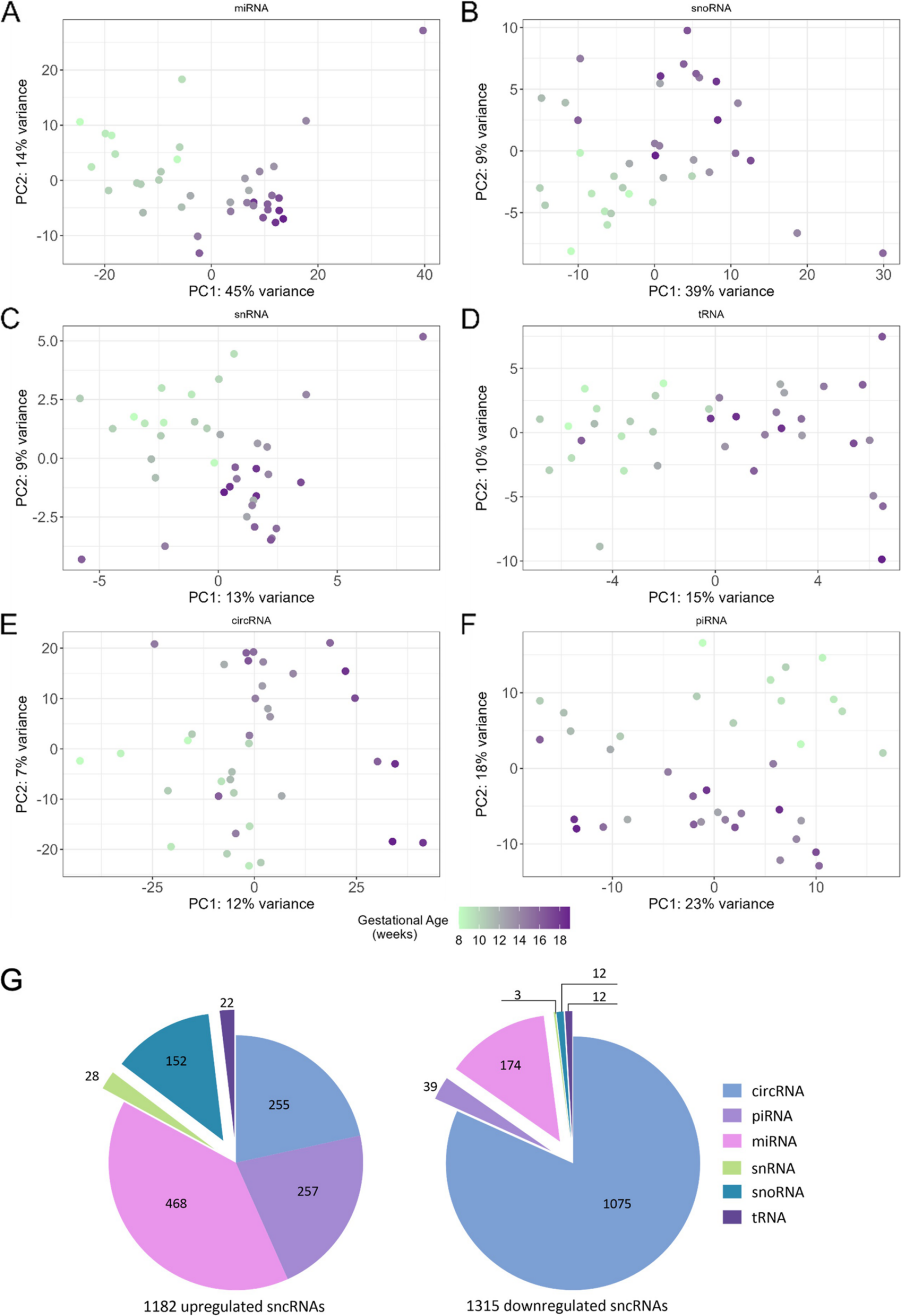
Small RNA populations showing differential expression during cardiac development. **A-F** Principal component analysis (PCA) plots displaying separation of each population of cardiac small RNA expression profiles by gestational age. **G** Pie charts displaying proportions of all small RNA populations upregulated (left) and downregulated (right) in the heart between weeks 8 and 19 of gestation

Analysis of each small RNA subtype expression profile revealed that variation of miRNAs, circRNAs, snoRNAs, snRNAs, and tRNAs each separated fetal heart samples by gestational age on PC1 or PC2 (Fig. 2A-F), suggesting that expression of all small RNA subtypes is developmentally regulated. Interestingly, variation in piRNA expression separated samples by gestational age on PC2 (Fig. 2F), suggesting that other factors could more strongly influence piRNA expression variability during heart development. Notably, PC1 explained the highest miRNA expression variation (45% variance) (Fig. 2A). In comparison, PC1 explained the smallest circRNA expression variation (12% variance) (Fig. 2E). Differential expression analysis of each small RNA subtype revealed that the largest fraction of upregulated small RNAs were miRNAs (39%), followed by piRNAs (22%) and circRNAs (22%), snoRNAs (13%), and tRNAs (2%) and snRNAs (2%) (Fig. 2G, Additional file 2, Additional file 3: Fig. 1SB-G). In contrast, the largest fraction of downregulated small RNAs were circRNAs (82%), followed by miRNAs (13%), piRNAs (3%), snoRNA (0.9%), tRNA (0.9%), and snRNA (0.2%) (Fig. 2G, Additional file 2, Additional file 3: Fig. 1SB-G). Taken together, these results suggest that all small RNA subtypes examined are differently up and downregulated in the heart as gestation proceeds, and that miRNA expression variability is most dependent on gestational age.

### miRNAs cluster within different expression patterns in the developing heart

**Fig. 3:**
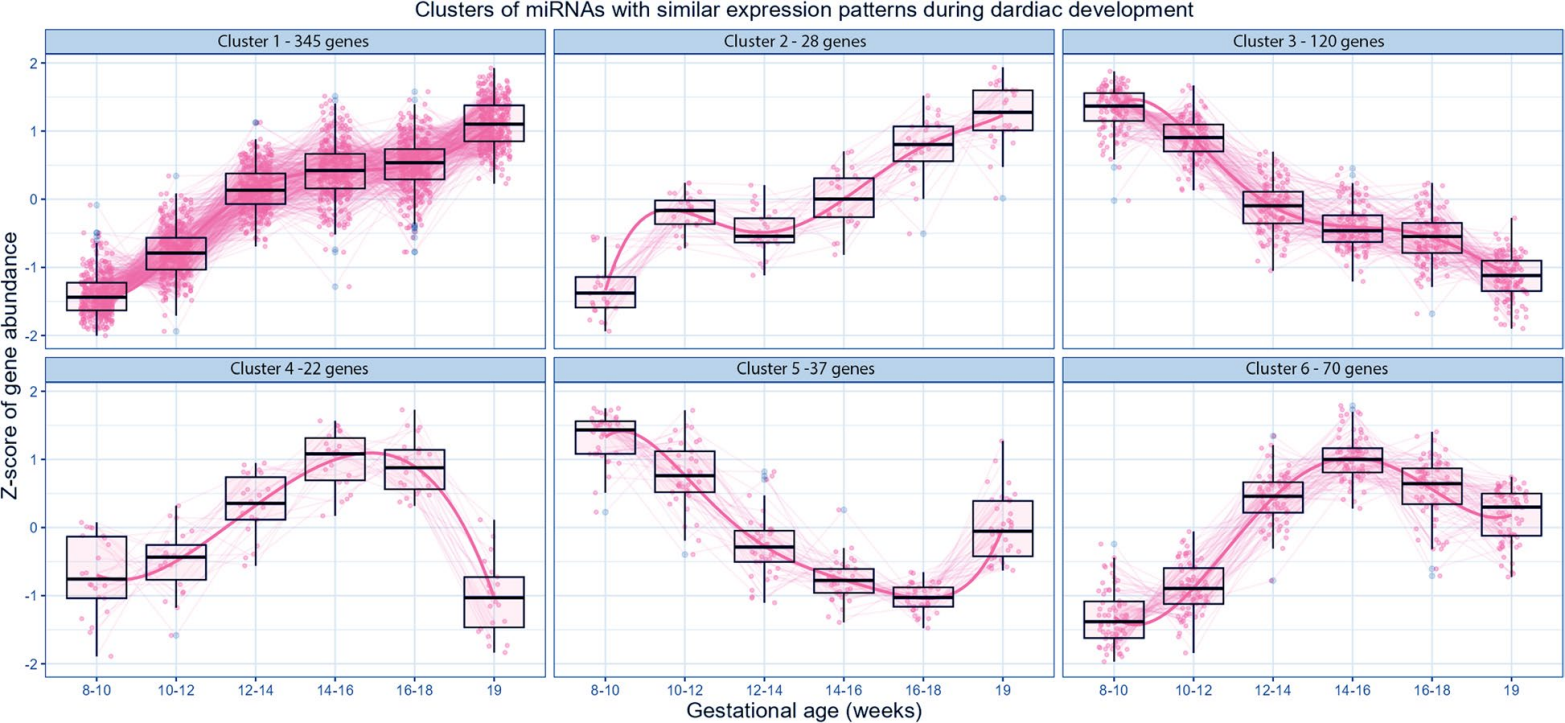
Clusters of miRNAs with Similar Expression Patterns during Cardiac Development. Scatter plots displaying the Z-score of gene abundance over gestational age for miRNA genes in each cluster of differentially expressed miRNAs with similar expression patterns (see Additional file 4) during cardiac development

642 miRNAs were dynamically expressed in the developing heart considering gestational age as a continuous variable (Fig. 2G, Additional file 4). Subsequent analysis to reveal miRNA expression patterns during mid-gestation identified six clusters among the 622 miRNAs. Such clusters included 22 to 345 miRNAs each (Fig. 3, Additional file 5). Only 20 miRNAs did not express in a distinguishable pattern and were excluded from subsequent analyses (see materials and methods).

To investigate the potential biological relevance of differentially expressed miRNAs, we performed functional enrichment analysis on each cluster (Additional file 3: Tables S1-S6). Most differentially expressed miRNAs clustered into clusters 1 and 2, which together included 373 miRNAs that were progressively upregulated during gestation (Fig. 3, Additional file 5). miRNAs in cluster 2 sharply increased from gestational week 10-12, briefly decreased between weeks 12 and 14, and then progressively increased until week 19 (Fig. 3). miRNAs in clusters 1 and 2 were most highly enriched for functional categories related to anti-cell proliferation, followed by immunity, cell differentiation and apoptosis (Additional file 3: Tables S1, 2). Cluster 3 included 120 miRNAs that were progressively downregulated as gestation progressed (Fig. 3). Such downregulated miRNAs were enriched for functional categories related to wound healing, myoblast differentiation, oxidative stress, and response to hypoxia (Additional file 3: Table S3). Clusters 4 and 6 consisted of 92 miRNAs each, exhibiting a more dynamic expression pattern, characterized by an initial increase in expression from gestational week 8 to 16, followed by a progressive decrease from week 16 to 19 of gestation (Fig. 3). Notably, miRNAs in cluster 4 were more drastically downregulated compared to those in cluster 6. miRNAs in cluster 4 were enriched for functional categories associated with muscle cell differentiation, immunity, and insulin resistance (Additional file 3: Table S4). On the other hand, miRNAs in cluster 6 were enriched for categories related to DNA damage response, plasma cell differentiation, immunity, and adiponectin signalling (Additional file 3: Table S6). miRNAs in cluster 6 (37 miRNAs) were expressed at lower levels at week 19, compared to week 8, and then were increased at week 19 of gestation (Fig. 3). miRNAs in cluster 5 were enriched for functional categories related to cellular differentiation, proliferation, and adiponectin signalling (Additional file 3: Table S6). Thus, miRNAs upregulated and downregulated in the heart during development could contribute to the regulation of cellular maturation, response to hypoxia, immunity, and metabolic processes. Notably, the majority (71%) of dynamically expressed miRNAs were upregulated (443 out of 622 miRNAs), while 179 miRNAs were downregulated across gestation. This suggests that miRNA expression is tightly regulated during fetal heart development, and that miRNAs could regulate different aspects of human heart maturation.

### mRNAs differentially expressed in inverse correlation with their targeting miRNAs control metabolic processes and cell cycle progression in the developing human fetal heart

miRNAs regulate the abundance of numerous mRNAs by targeting them for degradation [18]. To ascertain the impact of dynamically expressed miRNA on the abundance of their mRNA targets in the heart, we cross-referenced miRNA expression levels with mRNA transcriptomes obtained by RNAseq on the ventricles of 53 human hearts between 8 and 19 weeks of gestation (Additional file 6) [14].

**Fig. 4:**
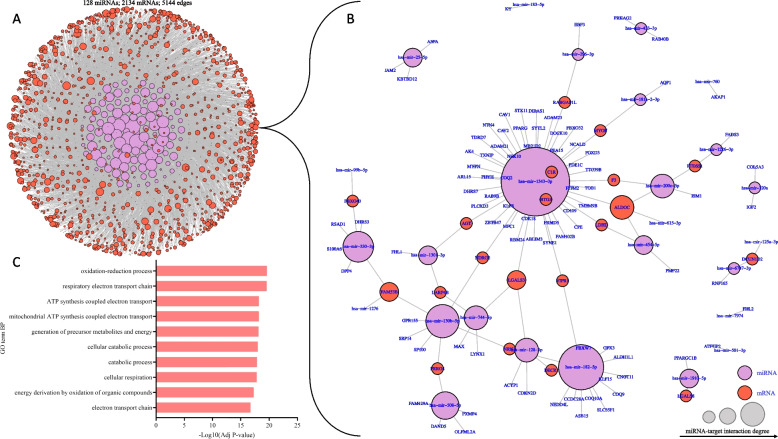
Integrative analysis of downregulated miRNAs and their upregulated mRNA targets. **A** miRNA-mRNA target interaction network depicting 5144 interactions (edges) between 128 differentially expressed miRNAs (purple nodes) and 2134 upregulated mRNA targets (red nodes). **B** A subset of the miRNA-mRNA target interaction network in (A) depicting the most significant negatively correlated pairs (Pearson correlation estimate < -0.7). **C** Top 10 gene ontology (GO) terms (biological functions) enriched amongst upregulated mRNA targets in fetal hearts between weeks 8 and 19 of gestation. Data was analyzed with DAVID v2023q1 using Benjamini correction. Abbreviations: *HADHA/B*, Hydroxyacyl-CoA dehydrogenase trifunctional multienzyme complex subunit alpha/beta*; CYC1*, Cytochrome c1; *UQCRC1*, Ubiquinol-cytochrome c reductase core protein 1; *ALDOC*, Aldose, fructose-bisphosphate C; ATP, Adenosine triphosphate

**Fig. 5:**
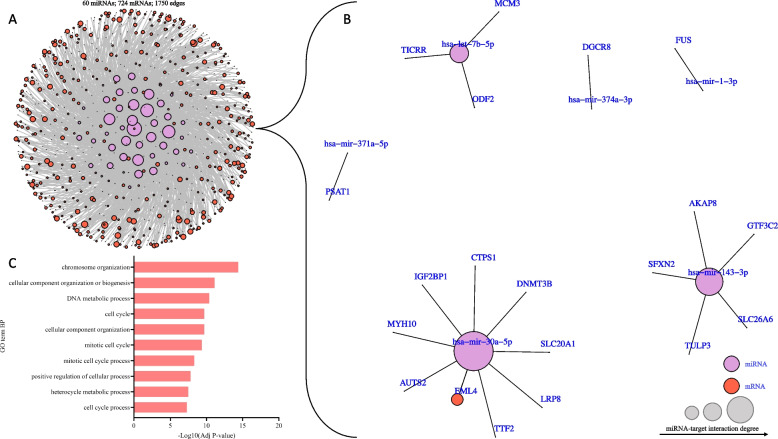
Integrative analysis of upregulated miRNAs and their downregulated mRNA targets. **A** miRNA-mRNA target interaction network depicting top 5% (1750 edges) negatively correlated 60 differentially expressed miRNAs (purple nodes) and downregulated mRNA targets (red nodes). **B** A subset of the miRNA-mRNA target interaction network in (A) depicting the most significant negatively correlated pairs (Pearson correlation estimate < -0.85). **C** Top 10 gene ontology (GO) terms (biological functions) enriched amongst downregulated mRNA targets in fetal hearts between weeks 8 and 18 of gestation. Data was analyzed with DAVID v2023q1 using Benjamini correction. Abbreviations: *EML4*, EMAP like 4; *TTF2*, Transcription termination factor 2; *DNMT3B*, DNA methyltransferase 3 beta; *AUTS2*, Activator of transcription and developmental regulator; *TICRR*, TOPBP1 interacting checkpoint and replication regulator; *MCM3*, Minichromosome maintenance complex component 3; *AKAP8*, A-kinase anchoring protein 8

As expected from a previous study [14], mRNA variation among samples separated human fetal hearts by gestational age on PC1 and sex on PC2, explaining 44 and 19% of the variations, respectively (Data not shown [41]). A total of 16,723 genes were differentially expressed (*P* value < 0.05) as gestation progressed, of which 8835 (53%) were upregulated, and 7888 (47%) were downregulated (Additional file 7). Upregulated genes were predominantly enriched for biological processes related to aerobic respiration and oxidative metabolism (Additional file 3: Fig. S2A), consistent with an energy metabolism shift during fetal heart development. In contrast, downregulated genes were enriched for biological processes related to cell morphogenesis, differentiation, and development (Additional file 3: Fig. S2B). Integrative analysis of differentially expressed miRNAs and predicted mRNA targets revealed that 128 downregulated miRNAs negatively correlate with 2134 upregulated mRNA targets (Additional file 3: Fig. S3A). Such targets predominantly control lipid and oxidative metabolism, specifically metabolic pathways involved in the electron transport chain and fatty acid metabolism (Fig. 4A-C, Additional file 3: Fig. S3B, C, Additional file 8). For example, *ALDOC*, which encodes Aldolase, Fructose-Biphosphate C, was predicted as a target of the downregulated *hsa-miR-1343 (MIR1343), hsa-miR454-5p (MIR454),* and *hsa-miR200c-3p (MIR200c),* and was upregulated as gestation progressed (Fig. 4B, Additional file 8). In contrast, 261 upregulated miRNAs negatively correlated with 4066 downregulated mRNA targets controlling cell cycle processes, DNA damage, replication, and repair (Additional file 3: Fig. S3D, E, Additional file 8). For example, *EML4*, which encodes EMAP Like 4, was predicted to be a target of the upregulated *hsa-miR-30a-5p (MIR30A)* and was downregulated as gestation progressed (Fig. 5B, Additional file 8).

To define the most important functions likely controlled by upregulated miRNAs, we focused on the 5% most negatively correlated miRNA-mRNA pairs. We found that 60 upregulated miRNAs correlated with the expression level of 724 significantly downregulated mRNAs (Fig. 5A, B). These downregulated mRNA targets, such as *EML4, TTF2, DNMT3B, AUTS2, TICRR, MCM3,* and *AKAP8,* were enriched for gene ontologies associated with cell cycle processes, including chromosome organization, mitosis, DNA metabolic processes and cell cycle phase transition, and correlated with the expression levels of *hsa-let-7b-5p (MIRLET7B), hsa-let-7f-5p (MIRLET7F1/2), hsa-miR-1-3p (MIR1-1/-2), hsa-miR-143-3p (MIR143), hsa-miR-146a-5p (MIR146A), hsa-miR-148b (MIR148B), hsa-miR-181d-5p (MIR181D), hsa-miR-21-5p (MIR21), hsa-miR-27b-3p (MIR27B), hsa-miR-30a-5p (MIR30A), hsa-miR-34c-5p (MIR34C), hsa-miR-378a-5p (MIR378A), hsa-miR-450b-5p (MIR450B), hsa-miR-455-5p (MIR455)* and *hsa-miR-542-3p (MIR542)* (Fig. 5C, Additional file 3: Fig. S4A, B, Additional file 8)*.* The overlap of enriched functions among mRNAs predicted as miRNA targets and differentially expressed mRNAs suggest that these miRNAs contribute to establishing the transcriptional landscape controlling metabolic shift and cell proliferation in the developing heart.

### Downregulated miRNAs negatively correlate with the abundance of proteins controlling aerobic metabolism in the developing heart

To obtain a more functional readout of dynamically expressed miRNAs, we examined global protein profiles obtained through liquid chromatography-tandem mass spectrometry (LC-MS/MS) on 10- and 18-week-old fetal hearts. A total of 340 and 407 proteins were detected across all three samples of 10- and three samples of 18- week-old fetal hearts, respectively (Additional files 9 and 10). Among these proteins, 248 were detected across all six samples analyzed, representing a subset of the approximately 2800 proteins previously detected in ventricles of human fetal hearts between gestational weeks 17 and 23 [48]. 72 proteins were differently abundant between the two gestational ages (*P* value < 0.05), of which 59 (83%) were enriched and 13 (17%) were depleted in 18-week-old hearts (Additional file 3: Fig. S5A).

**Fig. 6:**
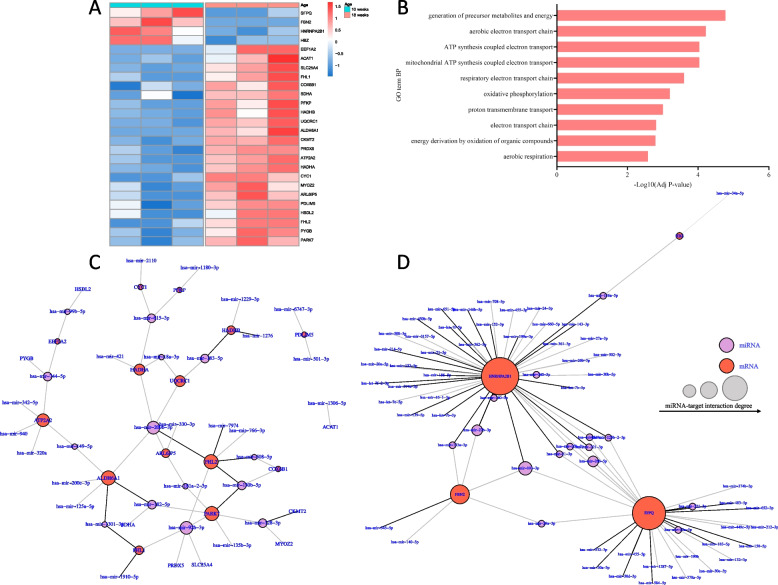
miRNA-mRNA-protein regulatory networks in fetal heart development. **A** Heatmap displaying normalized abundance (Z-scores) of differently abundant proteins identified as miRNA targets between 10- (*n* = 3) and 18-weeks-old (*n* = 3) fetal heart samples (*P* < 0.05). Enriched proteins are in red, and depleted proteins are in blue. **B** GO (gene ontology) biological processes enrichment among enriched proteins whose expression negatively correlates with miRNAs differential expression. GO enrichment was analyzed using g:profiler. **C** miRNA-mRNA targets-proteins interaction network depicting 58 interactions (edges) between 32 differentially expressed miRNAs (purple nodes) and 22 enriched proteins (red nodes) between 10- and 19-week-old fetal hearts. **D** miRNA-mRNA targets-proteins interaction network depicting 85 interactions (edges) between 66 differentially expressed miRNAs (purple nodes) and 4 depleted proteins (red nodes) between 10- and 18-week-old fetal hearts. Abbreviations: *SFPQ*, Splicing factor proline and glutamine-rich, *HNRNPA2B1*, Heterogeneous nuclear ribonucleoprotein A2/B1; *FBN2*, Fibrillin 2; *HBZ*, Hemoglobin subunit zeta

To uncover proteins whose abundance is regulated by miRNAs, we searched for differently abundant proteins encoded by mRNA targets whose levels were negatively correlated with miRNAs. Four proteins encoded by mRNAs that were decreased and predicted to be targeted by miRNAs were depleted, while 22 proteins encoded by upregulated mRNAs were enriched in 18-week-old hearts (Fig. 6A, Additional file 11).

Integration and functional enrichment analysis of data obtained from small RNA, mRNA and protein omics revealed a network of 32 downregulated miRNAs negatively correlated with 22 enriched proteins. Such proteins function in oxidative metabolism pathways and aerobic respiration (Fig. 6B, C). For example, the two subunits of the Mitochondrial trifunctional protein (MTP), HADHA (hydroxyacyl-CoA dehydrogenase trifunctional multienzyme complex subunit alpha) and HADHB (hydroxyacyl-CoA dehydrogenase trifunctional multienzyme complex subunit beta) [49, 50], and subunits of the Ubiquinol-Cytochorme c oxidoreductase (mitochondrial complex III), CYC1 (cytochrome c1) and UQCRC1 (ubiquinol-cytochrome c reductase core protein 1) [51], were highly enriched in 18-week-old fetal hearts and upregulated on the mRNA level across gestational age (Fig. 6A). The corresponding upregulated mRNAs were predicted as targets of the downregulated *hsa-miR-2110 (MIR2110), hsa-miR-615-3p (MIR615), hsa-miR-1276 (MIR1276), hsa-miR-183-5p (MIR183), hsa-miR-1229-3p (MIR1229), hsa-miR-1343-3p (MIR1343), hsa-miR-200b-3p (MIR200B), hsa-miR-421 (MIR421)* and *hsa-miR-18a-3p (MIR18A)* (Fig. 6C, Additional file 8).

In contrast, 66 upregulated miRNAs correlated with four depleted proteins (Fig. 6D). Although no significantly enriched functional pathways were associated with such depleted proteins, they are relevant for RNA processing and tissue homeostasis. Specifically, SFPQ (Splicing factor proline and glutamine-rich) and HNRNPA2B1 (Heterogeneous nuclear ribonucleoprotein A2/B1), are RNA-binding proteins (RBPs) that play crucial roles in RNA processing and splicing [52, 53]. Notably, SFPQ is involved in the production of circRNA [52], most of which were downregulated during gestation (Fig. 2G). Moreover, the extracellular matrix glycoprotein FBN2 (Fibrillin-2) is involved in the initial assembly of the aortic matrix of the heart [54]. These results suggest a function of miRNAs controlling the abundance of proteins regulating maturation of the human heart.

### miRNAs coordinate enrichment of components of the electron transport chain and AMP-activated signalling

**Fig. 7:**
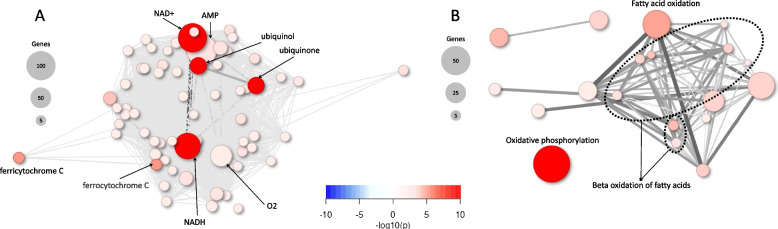
Reporter metabolite analysis of differentially expressed miRNA-predicted mRNA targets. Network plots of top 69 reporter metabolites (**A**) and top 20 metabolic reactions (subsystems) (**B**) identified analyzing genes differentially expressed in the heart from week 8 to 18 of gestation. Upregulated metabolites/metabolic reactions are in red, and downregulated metabolites/metabolic reactions are in blue. Analysis was performed with piano using distinct-directional adjusted *P* values with a cut-off of 0.01. Abbreviations: NAD+/NADH, Nicotinamide adenine dinucleotide; AMP, Adenosine monophosphate

**Fig. 8:**
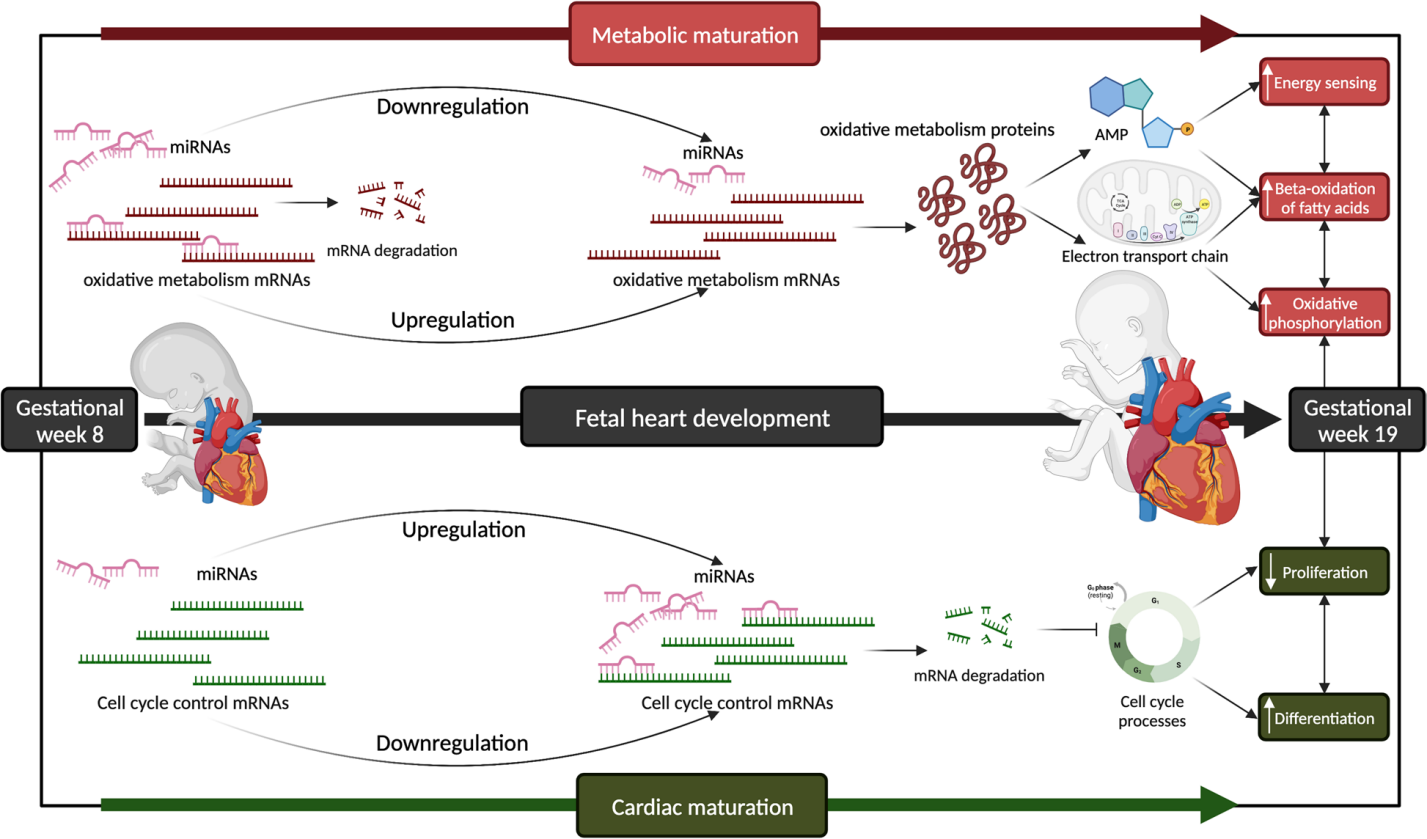
Working model of the function of miRNA-mRNA-protein networks in human fetal heart development. (Top panel) In the fetal heart at gestational week 8, key miRNAs mediate degradation of mRNAs encoding proteins regulating oxidative metabolism. As gestation progresses to 19 weeks, such miRNAs are downregulated, leading to the enrichment of target mRNAs and their encoded proteins thus allowing metabolic maturation of the heart as it adapts to progressive workload increase. Such proteins mediate an increase in AMP and enrichment of electron transport chain components, thereby boosting the heart’s capacity of fatty acids beta-oxidation and oxidative phosphorylation. (Bottom panel) In the fetal heart at gestational week 8, miRNAs that target mRNAs involved in cell cycle control are downregulated, preserving cardiac proliferation in the growing heart. As fetal development progresses and the heart matures, such miRNAs are upregulated, facilitating a progressive decrease in the proliferative capacity of cardiomyocytes. Figure was created with BioRender.com

To reveal metabolic processes regulated by miRNAs in the heart as it develops, we performed reporter metabolite analysis using the differentially expressed mRNAs predicted as miRNA targets. Reporter metabolite analysis predicts metabolite changes by linking differentially expressed genes to metabolic reactions [55]. We used two gene set collections to investigate the metabolic changes in the developing heart, one set containing metabolite-gene associations (Additional file 12) and the other containing metabolic pathway-gene associations (Additional file 13) [56]. A total of 229 of 705 metabolites were predicted to change (adjusted *P* < 0.05) in the heart as gestational age progressed. Notably all these metabolites were predicted to increase (Additional file 14). The largest clusters included components of the electron transport chain NAD+, NADH, ubiquinol, ubiquinone, ferricytochrome C, ferrocytochrome C and O2, as the most highly enriched metabolites (Fig. 7A). Interestingly, an additional large cluster of increased metabolites (115 mRNAs) was linked to AMP (Fig. 7A), which mediates metabolic sensing, glucose uptake and fatty acid oxidation through the AMP-activated protein kinase (AMPK) signalling pathway [57]. Likewise, at the subsystem level, the largest cluster including the most drastically increased metabolites was linked to oxidative phosphorylation (42 mRNAs) and fatty acid oxidation (31 mRNAs) (Fig. 7B, Additional file 15). To assess the specificity of the metabolite reporter networks controlled by miRNAs, we conducted metabolite reporter analysis on three additional control groups. These groups included the 10,533 differentially expressed mRNAs that were not identified as miRNA targets (Additional file 3: Fig. S6A, Additional file 16), 6200 randomly selected differentially expressed genes (Additional file 3: Fig. S6B, Additional file 16), and 6200 randomly selected differentially expressed genes that were also not predicted as miRNA targets (Additional file 3: Fig. S6C, Additional file 16). In contrast to clusters associated with miRNA targets, the predominant clusters of upregulated reporter metabolites in all three control groups were H+, ATP, ADP, H_2_O, and Pi/PPi. This suggests that most differentially expressed non-target mRNAs primarily influence the ADP-ATP cycle, further supporting a specific function of miRNA targets regulating the electron transport chain through NAD+ metabolism and AMP signaling. Moreover, at the subsystem level, the predominant cluster of metabolites associated with randomly selected non-target genes was linked to oxidative phosphorylation rather than fatty acid oxidation, which was linked with miRNA targets (Fig. 7B, Additional file 3: Fig. S6D). Interestingly, *HADHA* and *AHDHB* were found amongst the gene clusters linked to AMP, NAD+, NADH and fatty acid oxidation (Additional file 14). This further supports the notion that targets of *hsa-miR-615-3p, miR-421, miR-1229-3p, miR-18a-3p, miR-200-3p, miR-1276* and *miR-183-5p* could control a metabolic shift towards increased fatty acid oxidation in the developing human heart. Therefore, the downregulation of such miRNAs and accumulation of their mRNA targets could establish a transcriptional network that governs energy sensing and oxidative metabolism by increasing mediators of the electron transport chain, AMP signalling, and fatty acid oxidation during human heart development (Fig. 8).

## Discussion

Growing evidence highlights the importance of controlled expression of small non-coding RNAs in cardiac development and disease. For example, the expression of many myomiRs changes in human heart disease and during cardiac development [36, 58–60], and circRNAs are dysregulated in human cardiomyopathies [23, 61–64]. However, the expression patterns and biological functions of small RNA species in the developing human heart are still poorly understood. Here, we studied the expression dynamics of miRNA, piRNA, circRNA, snoRNA, snRNA, and tRNA in the heart of human fetuses at different time points during mid-gestation, providing insight into the molecular processes governing human fetal heart development.

We found that miRNAs were the most dynamically expressed small RNAs in the human heart as its develops (Figs. 1C, and 2A, G). circRNAs were notably downregulated over time, but such a trend was less dependent on gestational age than other small RNAs (Fig. 1A, C, Fig. 2E, G). In contrast, the expression of snoRNA and piRNA was less variable but was primarily explained by age (Fig. 2B, F, G). Our analysis focused on the function of miRNAs. Yet, all small RNA subtypes were dynamically expressed. This suggests that additional factors, such as sex or environmental changes, which were not considered in our analyses, could influence the expression of small RNAs. Indeed, sex significantly impacts the transcriptional landscape of the developing human heart [14, 65].

Cardiac and muscle-specific miRNAs have been primarily investigated for their functions in cardiomyocyte regeneration. Several miRNAs are positive and negative regulators of cardiomyocyte proliferation [66–70], differentiation [42, 71–74] and reprogramming [75–78]. We found six dynamic patterns of miRNA expression in the heart across gestation (Fig. 3). In agreement with previous studies [20–22, 39, 42, 66, 70–73, 78, 79], analysis of each pattern cluster revealed that miRNAs that are broadly upregulated as gestation proceeds have anti-proliferative activity and promote muscle cell differentiation (Fig. 3). In contrast, downregulated miRNAs play a role in response to hypoxia and oxidative stress. This suggests a potential function of miRNAs as regulators of cardiac maturation, driving progressive loss of cardiomyocyte proliferative capacity while promoting metabolic adaptation of the heart to an increasing oxygen demand of the growing fetus.

miRNAs often mediate reduced translation of target mRNAs [27]. Therefore, to reveal regulatory networks controlled by miRNAs during fetal heart development, we identified target mRNAs based on several criteria, including predicted targets (miRNet 2.0 target prediction tool [80]), the predicted target mRNA expression level, and the inverse correlation with their targeting miRNAs. Our findings suggest that mRNAs that are significantly downregulated in the heart and whose expression inversely correlates with targeting miRNA are involved in cell cycle progression-related functions, including cell cycle phase transition, DNA metabolic pathways, chromatin organization and mitosis (Fig. 5). These findings are consistent with miRNAs targeting mRNAs controlling cell cycle to limit or promote cell proliferation [81–86]. The upregulation of miRNAs with functions related to anti-proliferation and anti-differentiation could suggest progressively decreased cardiomyocyte proliferation as the fetal heart matures (Fig. 8).

In alignment with prior investigations, we observed upregulation of several members of the let-7 family (*let-7b/c/d/f/g/i*) [45, 87], the myomiRs *miR-1* [45], *miR-133a* [45] and *miR-499a* [45] in the heart across midgestation (Fig. 4A, B, Additional file 4, Additional file 8). Let-7 family plays a pivotal role in cardiovascular diseases [88], including dilated cardiomyopathy (DCM) [89], heart failure [46] and fibrosis [90] and are involved in cardiac embryonic stem cell differentiation and normal heart development [88, 91–93]. *miR-1* and *miR-133a* are specifically expressed in cardiac and skeletal muscle precursor cells during development [35, 38, 39], regulating muscle cell differentiation and proliferation [71]. Moreover, previous studies showed that the overexpression of *miR-1* and *miR-499* reduces the proliferative capacity of cardiomyocytes and are both enriched in differentiated cardiomyocytes [73].

Our network analysis also identified several miRNAs known to regulate cell cycle and proliferation that have yet to be extensively investigated in heart development. For example, *miR-30a* has been reported to reduce cancer cell proliferation by inhibiting cell cycle progression at the G0/G1 and G1/S transitions [94–97]. Thus, the expression of such miRNA in the fetal heart might have a similar function in influencing the cell cycle and promoting a loss of proliferative potential.

Shortly after birth, cardiomyocytes exit the cell cycle and lose their proliferative capacity in parallel with an increase in the mitochondrial oxidative capacity of the heart [11, 98, 99]. We found that mRNAs that are targeted by downregulated miRNAs, and their protein products, were the most substantially enriched in the fetal heart as it develops and regulates oxidative metabolism and aerobic respiration (Fig. 4), Fig. 6A-C. The heart grows in a state of relative hypoxia, which is critical for proper myocardium formation and maturation [3, 7, 100]. The fetal heart metabolism is remarkably flexible, switching from aerobic to anaerobic respiration and increased glycolysis in response to low oxygen tension [101]. However, prolonged exposure to hypoxia can be detrimental and even make the heart susceptible to cardiomyopathy later in life [102–109]. The postnatal heart grows by hypertrophy to adapt to the increased workload and increase its oxidative capacity [10, 110]. Therefore, the miRNA-mRNA inverse-correlation and upregulation of genes controlling oxidative metabolism suggest that miRNA networks could regulate the metabolic shift of the heart during fetal development (Fig. 8). Energy homeostasis in the heart requires balanced anaerobic and aerobic metabolism [111, 112]. Our reporter metabolite analysis suggests that miRNA-mRNA regulatory networks are linked to the increase of metabolites that are critical electron carriers of the electron transport chain, including NAD+ and ubiquinone (Fig. 7A). Notably, AMP, an activator of the AMP-associated protein kinase (AMPK) pathway (Fig. 7A) was also predicted to increase. AMPK senses cellular energy status and regulates its homeostasis [57]. Mounting evidence indicates that miRNAs control AMPK signalling and influence cardiac metabolism and cardiovascular disease [113–119]. For instance, in naked mole-rats, miRNAs inhibit AMPK to coordinate the downregulation of skeletal muscle-specific metabolism under hypoxia [115]. In diabetic cardiomyopathy, specific miRNAs suppress AMPK, leading to increased lipotoxicity in cardiac myocytes and promoting diet-induced cardiac hypertrophy in mice [119]. Conversely, inhibition of miRNA-27b in brain microvascular endothelial cells (BMECs) and mouse ischemic stroke models activates AMPK, promoting angiogenesis and facilitating post-stroke recovery [116].

Our findings suggest that specific miRNAs, including *miR-1276, miR-183-5p*, and *miR-1229*, regulate the expression of *HADHB*, while *miR-615-3p, miR-421, miR-200b-3p,* and *miR-18a-3p* regulate the expression of *HADHA*. This leads to the accumulation and translation of these miRNAs into proteins that control the metabolic adaptation of the growing fetal heart. Indeed, such a miRNA-mRNA-protein regulatory network could control the upregulation of fatty acid oxidation and oxidative phosphorylation by increasing AMP and critical electron carriers of the electron transport chain (Fig. 7). Thus, the downregulation of these key miRNAs could facilitate increasing the capacity of the heart to utilize fatty acids and rely more on oxidative metabolism as it prepares to transition from the hypoxemic intrauterine environment to the postnatal oxygen-rich environment (Fig. 8). Notably, *miR-183* has previously been shown to modulate the tricarboxylic acid (TCA) cycle in glioma cells [120, 121]. In addition, upregulation of *miR-183* in lung cancer cells decreased mitochondrial oxygen consumption and ATP production, inhibiting cell proliferation [122]. Interestingly, a lower abundance of *miR-183* is significantly associated with worsening heart failure in transposition of the great artery (TGA) patients [123] and rats with chronic systolic heart failure [124], suggesting a plausible role of *miR-183* in maintaining mitochondrial energy metabolism homeostasis during heart development.

Multiple miRNAs can bind at imperfect recognition sites, cooperatively fine-tuning the translation of an mRNA [125–127]. This suggests redundant functions for multiple miRNAs [128]. Notably, a higher number of miRNAs were upregulated in the fetal heart. However, downregulated miRNAs were more clearly linked to an mRNA-protein network controlling metabolism. Thus, miRNAs suppressing metabolism must be downregulated to allow metabolic maturation of the fetal heart.

Our study integrated the analysis of transcriptomes and proteomes of the human developing heart. Both approaches provide valuable insight, but each has limitations. While transcriptome analysis offers an overview of dynamic gene expression, mRNA levels do not always correlate with protein abundance due to posttranscriptional modifications, alternative splicing, and protein degradation [129, 130]. Additionally, bulk RNA sequencing of ventricular tissue encompasses multiple cell types, including fibroblasts, smooth muscle and endothelial cells, which may impact the observed expression patterns. Proteomics provides more direct functional insight [131]. However, proteomics’ limited dynamic range and detection sensitivity challenge the detection and quantification of low-abundance proteins, providing limited coverage of the overall protein profile [130, 131]. Our proteomics analysis included six samples at two gestational ages, whereas our transcriptomic analysis included 53 samples at multiple gestational ages. Accordingly, downregulated mRNAs poorly matched with proteins depleted over gestational age. This suggests that our analysis likely predicted a small fraction of miRNA-mRNA-protein interactions. Proteomics on a larger number of hearts at more gestational stages is required to better understand the function of miRNAs, for example, in suppressing proliferation potential as reflected by mRNA but not protein profiles.

Moreover, a large number of differentially expressed mRNAs (*n* = 16,723 genes) that were not predicted as miRNA targets (*n* = 10,534 genes) were not further analyzed. This raises the possibility that unknown miRNAs or perhaps other small RNA types could contribute to shaping the mRNA landscape of the developing heart. Despite that functional pathways were not enriched among depleted proteins inversely correlated with upregulated miRNAs, one depleted protein, SFPQ (Splicing factor proline and glutamine-rich), is a transcriptional activator that regulates the production of circRNAs [52]. Interestingly, circRNA was the most prominently downregulated small RNA subtype (Fig. 1F). Thus, additional small RNA networks, perhaps a miRNA-circRNA-mRNA pathway, might operate in human heart development.

## Conclusions

In this study, we uncovered patterns of small RNA expression in the human developing heart. Moreover, we link dynamically expressed miRNAs to mRNAs and proteins regulating cell proliferation and metabolism as the heart develops, revealing candidate miRNAs maintaining cardiac homeostasis. Downregulated mRNAs predicted to be targeted by miRNAs upregulated during gestation control cell cycle processes, potentially influencing cardiac growth and maturation. In contrast, upregulated mRNAs predicted to be targeted by miRNAs downregulated during gestation could control energy homeostasis, increasing energy sensing and oxidative metabolism. Thus, our results suggest that a miRNA-mRNA-protein network regulates growth and metabolic maturation of the human fetal heart during mid-gestation.

## Methods

### Human fetal heart samples

Fetal heart samples were obtained from the Research Center for Women and Infant Health (RCWIH) BioBank (Mount Sinai Health System, Toronto, Ontario, Canada), and studies were approved by the ethics Research Boards at the Hospital for Sick Children and Mount Sinai Hospital. Samples were collected from healthy donors undergoing elective pregnancy termination, and terminations due to congenital heart defects were excluded [14]. Informed consent for human sample collection was provided by all donors. Fetal hearts were dissected and frozen in RNAlater Stabilization Solution (Thermo Fisher Scientific) at -80 °C.

### Sample preparation and RNA extraction

For sample preparation, whole hearts were stored at -80 °C in RNAlater Stabilization Solution (Thermo Fisher Scientific). After thawing, ventricles were isolated from the atria and major vessels. To eliminate blood inside the chambers, the ventricles were cut open, rinsed with PBS for 10 seconds and kept in TRIzol. Ventricles were then homogenized at 30 kHz for 1 min using TissueLyser II (Qiagen) and centrifugated to pellet the insoluble tissue. DNA-free RNA was extracted using a Direct-zol RNA MiniPrep kit (Zymo Research).

### Small RNA library preparation and sequencing

Small RNA libraries were prepared using the NEBNext Small RNA Library Prep Kit for Illumina with 1 μg total RNA starting material according to published protocol (NEBNext® Multiplex Small RNA Library Prep Set for Illumina® Set 1, Set 2, Index Primers 1–48 and Multiplex Compatible). Single-end RNA sequencing (50 bp) was performed on an Illumina Hi-Seq 2500 platform.

### Small RNA-seq data analysis

For pre-processing and annotation, raw sequence reads were trimmed, poor quality reads were removed, reads were aligned and annotated with COMPSRA [132]. Sequences were aligned to the human genome (hg38), and reads were annotated using several small RNA reference databases (miRbase [133], gtRNAdb [134], gencode [135], circBase [136] piRNABank [137], piRBase [138] and piRNACluster [139]). Reads mapped to multiple genes were removed from further analysis and only single mapped genes were retained to avoid ambiguous results in downstream analyses, rendering a total of 40,229 annotated small RNAs (39,402 non-zero genes) (Additional file 17). Differential expression analysis was performed on R 4.0.4 with DESeq2 v1.30.1 [140]. Adjusted *P* values < 0.05 were considered significant and no Log_2_ FC cutoff was applied. PCA plots were generated using ggplot2 3.4.0 and heatmaps were generated with pheatmap 1.0.12 in R 4.0.4. The small RNA-seq dataset is available in the NCBI GEO repository under accession number GSE241759.

### miRNA expression clustering and functional analysis

Z-score values of miRNA gene expression corresponding to each gestational age range (8-10, 10-12, 12-14, 14-16, 16-18, 19) were calculated using R 4.0.4. Significant differentially expressed miRNA genes were clustered with DegPatterns on R 4.0.4 using calculated Z-scores of expressions and a minimum of 15 genes per cluster. Functional enrichment analysis of miRNAs in each cluster was performed with miRNet 2.0 [80].

### RNA-seq data analysis

Mapped sequencing reads were obtained from transcriptomic profiling of fetal hearts (*n* = 53) previously published [14]. Total counts were quantified using FeatureCounts and normalized with batch correction using DESseq2 version 1.30.1 [140]. Adjusted *P* value *< 0.05* was considered significant and no Log_2_ FC cutoff was applied. PCA plots were generated using ggplot2 3.4.0 and heatmaps were generated with pheatmap 1.0.12 in R 4.0.4. Gene Ontology (GO) term enrichment was performed using DAVID v2023q1 [141] with Benjamini correction or g:profiler [142] using default settings. Bar graphs were created using Prism 9.5.1. The RNA-seq dataset is available in the NCBI GEO repository under accession number GSE241758.

### Inverse-correlation of miRNA and mRNA expression data

Target prediction and miRNA-mRNA pairwise interaction of differentially expressed miRNAs was performed using miRNet2.0 target prediction tool [80]. We overlapped mRNA targets with differentially expressed mRNAs and obtained pairwise interactions between differentially expressed miRNAs and differentially expressed mRNA targets. Pearson correlation was then performed between differentially expressed miRNAs and mRNAs using normalized counts, and significant and anti-correlated pairs were retained *(P* value < 0.05 and *r* < 0). Significant negatively correlated DEmiRNA-DEmRNA pairs were grouped based on Log_2_FC of mRNA expression (Log_2_ FC > 0; Log_2_ FC < 0). Interaction network graphs were generated with iGraph 1.3.0 [143] on R 4.0.4 using layout on sphere.

### Protein sample preparation and liquid chromatography-tandem mass spectrometry (LC-MS/MS)

For proteomic analysis, we used 6 whole frozen ventricles from 10- and 18- weeks fetal hearts (*n* = 3; n = 3). To achieve efficient lysis and protein extraction from the tissue samples, ventricles were minced, washed with PBS, and homogenized in RIPA lysis and extraction buffer (Thermo Fisher Scientific) followed by mechanical lysis through sonication for a total of 3 minutes and then centrifugation at 25,000 g for 1 hour at 4 °C to remove DNA, RNA, and other cell debris. Protein supernatants were retained, and proteins were digested following an in solution tryptic digestion protocol (Additional file 18). Briefly, samples were resuspended in 50 mM Ammonium Bicarbonate digestion buffer to a total volume of 100 μL. Next, 5 μL of DTT (200 mM DTT in 100 mM ammonium bicarbonates) was added and protein solutions were boiled for 10 minutes. Next, 8 μL of iodoacetamide (0.5 M iodoacetamide in 100 mM ammonium bicarbonate) was added, and protein solutions were kept in the dark at room temperature for 45 minutes. Then, another 20 μL of DTT was added and samples were left at room temperature for 45 minutes. Lastly, 1 μL of trypsin was added to samples followed by overnight incubation at 37 °C. The samples were sent for Liquid chromatography coupled with tandem mass spectrometry (MS/MS) analysis and data pre-processing at the BioZone Mass Spectrometry Facility in the Chemical Engineering Department at the University of Toronto. Samples were analyzed using a nanoLC Q-Exactive Mass Spectrometer with C18 column (Thermo Scientific, Waltham, MA, USA). Water and acetonitrile containing 0.1% formic acid were used as eluents. Each sample was analyzed independently for 120 minutes (see Additional file 18). The mass spectrometry proteomics data have been deposited to the ProteomeXchange Consortium via the PRIDE partner repository with the dataset identifier PXD044936.

### Proteomics data analysis and mRNA-protein integration

Raw peaks were pre-processed (raw signals exacting, data baselines filtering, peak identification, and integration) using GPMDB (Global Proteome Machine and Database) with x!Tandem algorithm (see Additional file 18). Common proteins identified across all samples were retained and *P* value < 0.05 was considered significant. Differentially abundant proteins were compared against differentially expressed mRNAs identified as miRNA targets. mRNA-protein pairs were considered significant if differential expression directionality was the same and *P* value < 0.05. Heatmaps were generated with pheatmap in R. Gene Ontology (GO) term enrichment was performed using DAVID v2023q1 [141] with Benjamini correction or g:profiler [142] using default settings. Bar graphs were created using Prism 9.5.1. Interaction network graphs were generated with iGraph 1.3.0 [143] on R using nicely layout option.

### Reporter metabolite analysis

The R package piano 2.6.0 [144] was used for reporter metabolite analysis. mRNA fold changes and adjusted *P* values of all mRNAs identified as miRNA targets (from DESeq2 and miRNA-mRNA correlation analysis) were used as input, and the genome-scale metabolic and metabolic reactions (subsystems) models from the Human-GEM v1.12.0 [56] were used as gene set collections (gsc) and downloaded from GSAM [145]. We used 1000 permutations, and a null distribution for significance assessment of gene sets. We set the smallest and largest gene set sizes allowed in the analysis to 5 and 500, respectively. To define a process or pathway as significant, we used an adj *P* < 0.05 as a cut-off for “distinct direction” of piano. For control groups, we used the “sample” function in R 4.0.4 to randomly select a group of differentially expressed genes.

### Supplementary Information

**Additional file 1.** Sample information of 37 fetal hearts used in the study.

**Additional file 2.** Expression values of differentially expressed small RNAs.

**Additional file 3: Fig. S1A-G.** Significant differentially expressed genes across gestational age (*P* value < 0.05) of each small RNA population. **Table S1-6.** Functional enrichment analysis of miRNAs clustered in each of six clusters of expression pattern. **Fig. S2.** Gene ontology terms (biological functions) and functional annotations (KW) enriched amongst genes upregulated (A) and downregulated (B) in fetal hearts across gestation. **Fig. S3.** (A) Analysis pipeline used to correlate miRNA expression data to mRNA expression data. (B, C) Functional enrichment analysis of differentially expressed miRNA-mRNA targets. **Fig. S4**A, B. Functional enrichment analysis of top 5% downregulated miRNA-mRNA targets. **Fig. S5.** Significant differentially expressed proteins between 10- and 18-weeks fetal hearts (*P* value < 0.05). **Fig. S6.** Controls for reporter metabolite analysis.

**Additional file 4.** Significant differentially expressed and uniquely mapped miRNAs.

**Additional file 5.** Cluster assignment of significant differently expressed miRNAs.

**Additional file 6.** Sample information of 53 fetal heart samples used in this study.

**Additional file 7.** Significant differentially expressed mRNA across gestation (*P* value < 0.05).

**Additional file 8.** Significant differentially expressed and negatively correlated miRNA-mRNA pairs.

**Additional file 9.** Abundance of proteins detected across all 3 samples of 10 weeks old fetal hearts.

**Additional file 10.** Abundance of proteins detected across all 3 samples of 18 weeks old fetal hearts.

**Additional file 11 **Significant differentially abundant proteins (*P* value < 0.05) identified as miRNA targets.

**Additional file 12.** Human-GEM derived get set collection metabolite level.

**Additional file 13.** Human-GEM derived get set collection subsystem level.

**Additional file 14.** Reporter metabolites analysis results (metabolites).

**Additional file 15.** Reporter metabolites analysis results (subsystems).

**Additional file 16.** Reporter metabolite analysis results for control gene sets. (6200 randomally selected mRNAs, 6200 random non-targets, all non-targets).

**Additional file 17.** Small RNA-seq expression profiles of uni-mapped genes annotated with COMPSRA.

**Additional file 18.** Full protocols for protein digestion and identification for mass spectrometry.

## Data Availability

All data generated during this study are included in this published article and its supplementary files. The RNA-seq and small RNA-seq datasets generated and analysed during the current study are available in the NCBI GEO repository, with accession numbers GSE241758 and GSE241759 (GEO SuperSeries: GSE241760). The mass spectrometry proteomics data have been deposited to the ProteomeXchange Consortium via the PRIDE partner repository with the dataset identifier PXD044936.

## Abbreviations

miRNA: MicroRNA
piRNA: Piwi-interacting RNA
circRNA: Circular RNA
snoRNA: Small nucleolar RNA
snRNA: Small nuclear RNA
tRNA: Transfer RNA
sncRNA: Small noncoding RNA
UTR: Untranslated region
PC: Principal component
ALDOC: Aldose, fructose-bisphosphate C
EML4: EMAP like 4
TTF2: Transcription termination factor 2
DNMT3B: DNA methyltransferase 3 beta
AUTS2: Activator of transcription and developmental regulator AUTS2
TICRR: TOPBP1 interacting checkpoint and replication regulator
MCM3: Minichromosome maintenance complex component 3
AKAP8: A-kinase anchoring protein 8
HADHA: Hydroxyacyl-CoA dehydrogenase trifunctional multienzyme complex subunit alpha
HADHB: Hydroxyacyl-CoA dehydrogenase trifunctional multienzyme complex subunit beta
CYC1: Cytochrome c1
UQCRC1: Ubiquinol-cytochrome C reductase core protein 1
LC-MS/MS: Liquid Chromatography-Tandem mass spectrometry
SFPQ: Splicing factor proline and glutamine rich
HNRNPA2B1: Heterogeneous nuclear ribonucleoprotein A2/B1
RBPs: RNA-binding proteins
FBN2: Fibrillin-2
NAD+/NADH: Nicotinamide adenine dinucleotide
AMP: adenosine monophosphate
AMPK: AMP-activated protein kinase
ATP: Adenosine triphosphate
ADP: Adenosine diphosphate
ETC: Electron transport chain
TCA cycle: Tricarboxylic acid cycle
