# Supplementary material for: Integrated small RNA, mRNA and protein omics reveal a miRNA network orchestrating metabolic maturation of the developing human heart
Source: BMC Genomics. 2023 Nov 23;24:709. doi: 10.1186/s12864-023-09801-8 (PMC10668469; doi:10.1186/s12864-023-09801-8)
Supplement: Supplementary file 3 — Additional file 3: Fig. S1A-G. Significant differentially expressed genes across gestational age (P value < 0.05) of each small RNA population. Table S1-6. Functional enrichment analysis of miRNAs clustered in each of six clusters of expression pattern. Fig. S2. Gene ontology terms (biological functions) and functional annotations (KW) enriched amongst genes upregulated (A) and downregulated (B) in fetal hearts across gestation. Fig. S3. (A) Analysis pipeline used to correlate miRNA expression data to mRNA expression data. (B, C) Functional enrichment analysis of differentially expressed miRNA-mRNA targets. Fig. S4A, B. Functional enrichment analysis of top 5% downregulated miRNA-mRNA targets. Fig. S5. Significant differentially expressed proteins between 10- and 18-weeks fetal hearts (P value < 0.05). Fig. S6. Controls for reporter metabolite analysis. [file 12864_2023_9801_MOESM3_ESM.pdf]

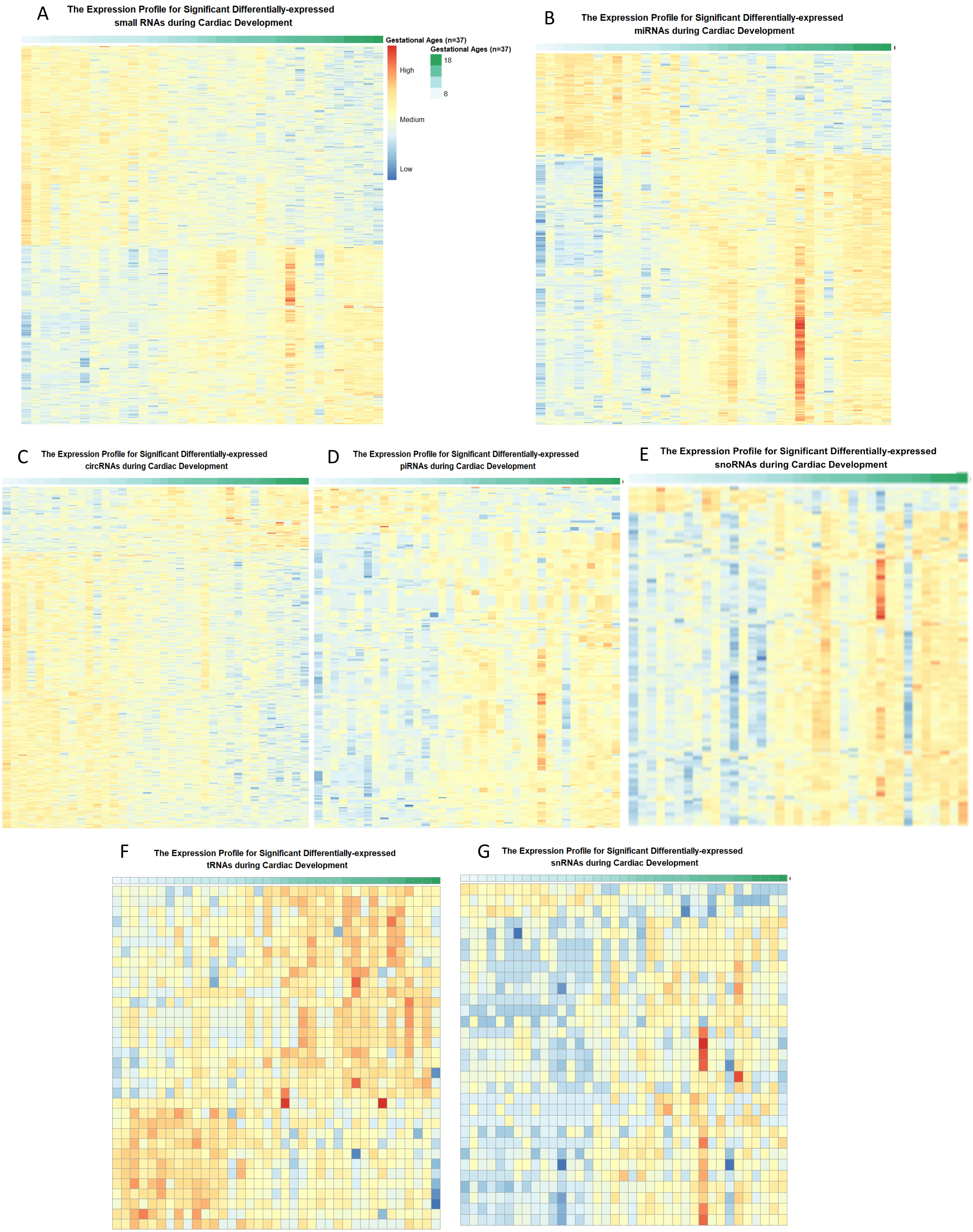

Figure S1. Significant differentially expressed small RNA species across gestational age. **(A-G)** Heatmaps displaying normalized expression (Z-score) of significantly deregulated small RNA genes across gestation (P value < 0.05).

Name	Hits	Pval	Adj.Pval
Anti-cell proliferation	21	5.24E-10	5.24E-08
Innate immunity	57	1.26E-07	0.0000039
Onco-MiRNAs	54	1.52E-07	0.0000039
Immune system	36	1.56E-07	0.0000039
Hematopoiesis	71	4.43E-07	0.00000886
Granulopoiesis	20	0.0000044	0.0000733
Smooth muscle cell differentiation	9	0.00000677	0.0000967
T-cell differentiation	27	0.0000102	0.000128
Skeletal muscle cell differentiation	32	0.0000259	0.000288
Nephrotoxicity	27	0.0000458	0.000428

Table S1. Top 10 enriched biological functions of miRNAs in cluster 1. Data was analyzed using miRNet2.0.

Name	Hits	Pval	Adj.Pval
Cytokine Secretion	3	0.000133	0.0045
Apoptosis	11	0.000155	0.0045
Immune System	6	0.000941	0.0182
Estradiol Synthesis	2	0.00369	0.0469
Adipogenesis	5	0.00404	0.0469
Regulation of GnRH Pathway	2	0.00547	0.0529
Peritoneal Cavity Homeostasis	5	0.00873	0.0723
Cell Growth	2	0.0127	0.0786
T-Cell Differentiation	4	0.0129	0.0786
Immune Response	8	0.0144	0.078

Table S2. Top 10 enriched biological functions of miRNAs in cluster 2. Data was analyzed using miRNet2.0.

Name	Hits	Pval	Adj.Pval
Wound Healing	9	0.0124	0.435
Myoblast Differentiation	3	0.0134	0.435
Oxidative Stress	5	0.0165	0.435
Cell Motility	8	0.025	0.435
Keratinocyte Apoptosis	2	0.0317	0.435
T-Cell Activation	4	0.0384	0.435
Antiviral Immunity	2	0.0458	0.435
Vascular Remodeling	2	0.0458	0.435
Response to Hypoxia	5	0.0481	0.435
Neurotoxicity	7	0.0489	0.435

Table S3. Top 10 enriched biological functions of miRNAs in cluster 3. Data was analyzed using miRNet2.0.

Name	Hits	Pval	Adj.Pval
Tumor Suppressor MiRNAs	11	0.000589	0.034
Ovarian Follicle Development	3	0.00103	0.034
Skeletal Muscle Cell Differentiation	5	0.0099	0.127
Circadian Rhythm	5	0.0106	0.127
Innate Immunity	7	0.0107	0.127
Cholesterol Metabolism	3	0.0141	0.127
Insulin Resistance	4	0.0172	0.127
Cholesterol Homeostasis	3	0.0196	0.127
Vascular Smooth Muscle Cell Differentiation	3	0.0196	0.127
Regulation of Akt Pathway	5	0.0204	0.127

Table S4. Top 10 enriched biological functions of miRNAs in cluster 4. Data was analyzed using miRNet2.0.

Name	Hits	Pval	Adj.Pval
Brain Development	9	0.000424	0.0237
Genomic Instability	3	0.00109	0.0305
Plasma Cell Differentiation	4	0.00205	0.0344
Neuron Differentiation	5	0.00246	0.0344
Endothelial Cell Proliferation	3	0.00773	0.0812
Keratinocyte Proliferation	2	0.0087	0.0812
Myofibroblast Differentiation	2	0.0199	0.159
Adiponectin Signaling	2	0.0349	0.244
Vascular Homeostasis	3	0.0405	0.252
Response to Hypoxia	3	0.0512	0.287

Table S5. Top 10 enriched biological functions of miRNAs in cluster 5. Data was analyzed using miRNet2.0.

Name	Hits	Pval	Adj.Pval
Histone Modifications	3	0.000269	0.0256
DNA Damage Response	8	0.01	0.437
Plasma Cell Differentiation	5	0.0138	0.437
Peritoneal Cavity Homeostasis	9	0.0277	0.658
Adiponectin Signaling	3	0.0386	0.733
Cytokine Secretion	2	0.0534	0.788
Natural Killer Cell Activation	3	0.0581	0.788
Oxidative Stress	4	0.0843	0.95
Cholesterol Homeostasis	4	0.0943	0.95
Hormone-mediated Signaling Pathway	15	0.113	0.95

Table S6. Top 10 enriched biological functions of miRNAs in cluster 6. Data was analyzed using miRNet2.0.

A

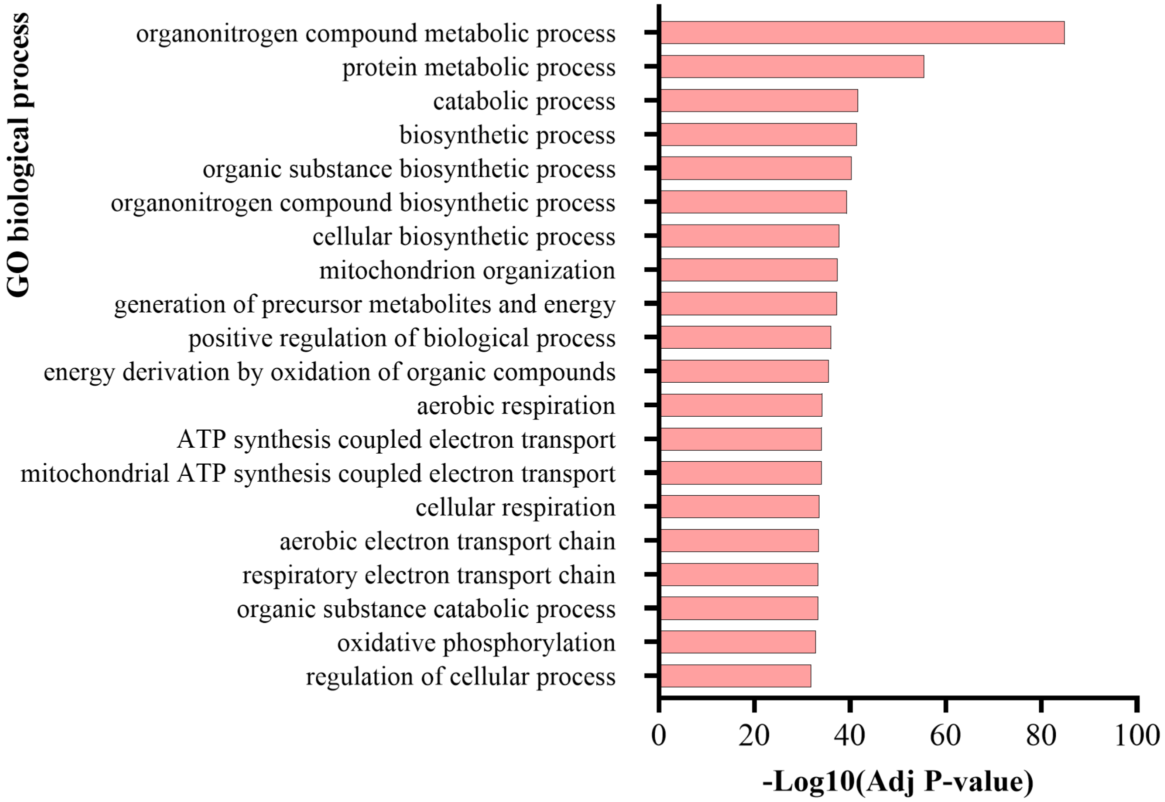

B

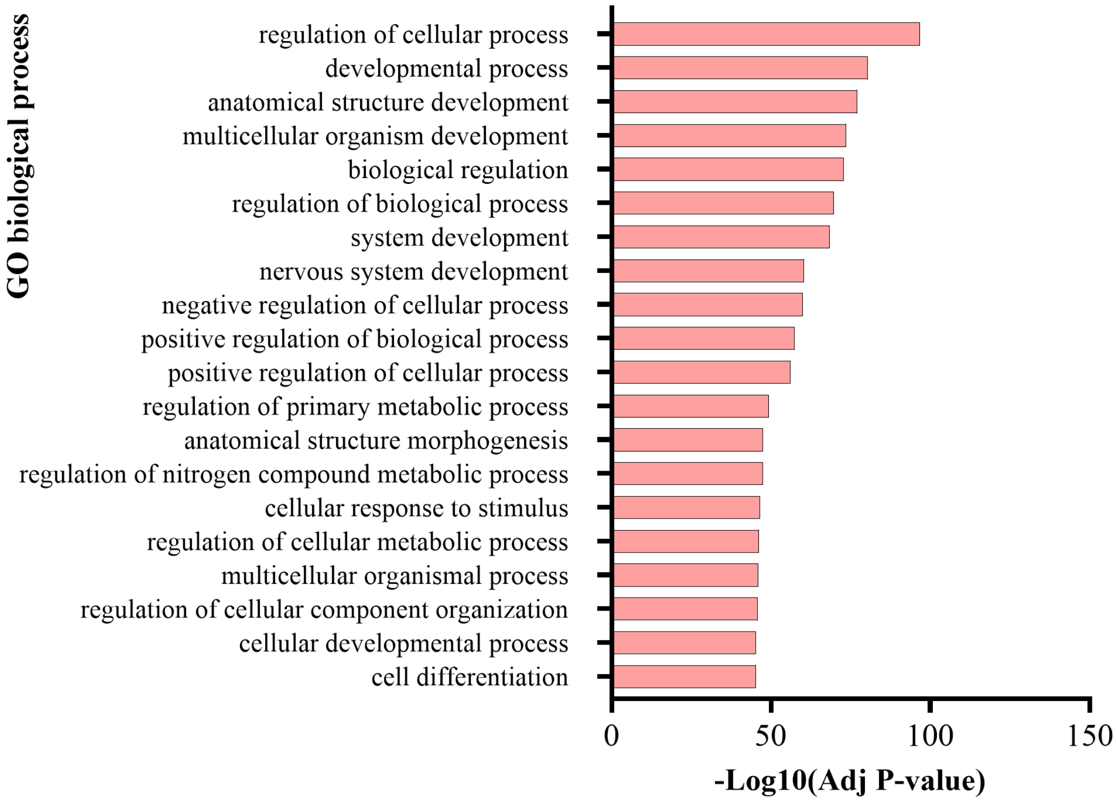

Figure S2. Top 20 gene ontology terms (biological functions) and functional annotation category (uniprot key word biological processes) enriched amongst genes upregulated **(A)** and downregulated **(B)** in fetal hearts across gestation. Data was analyzed using g:profiler.

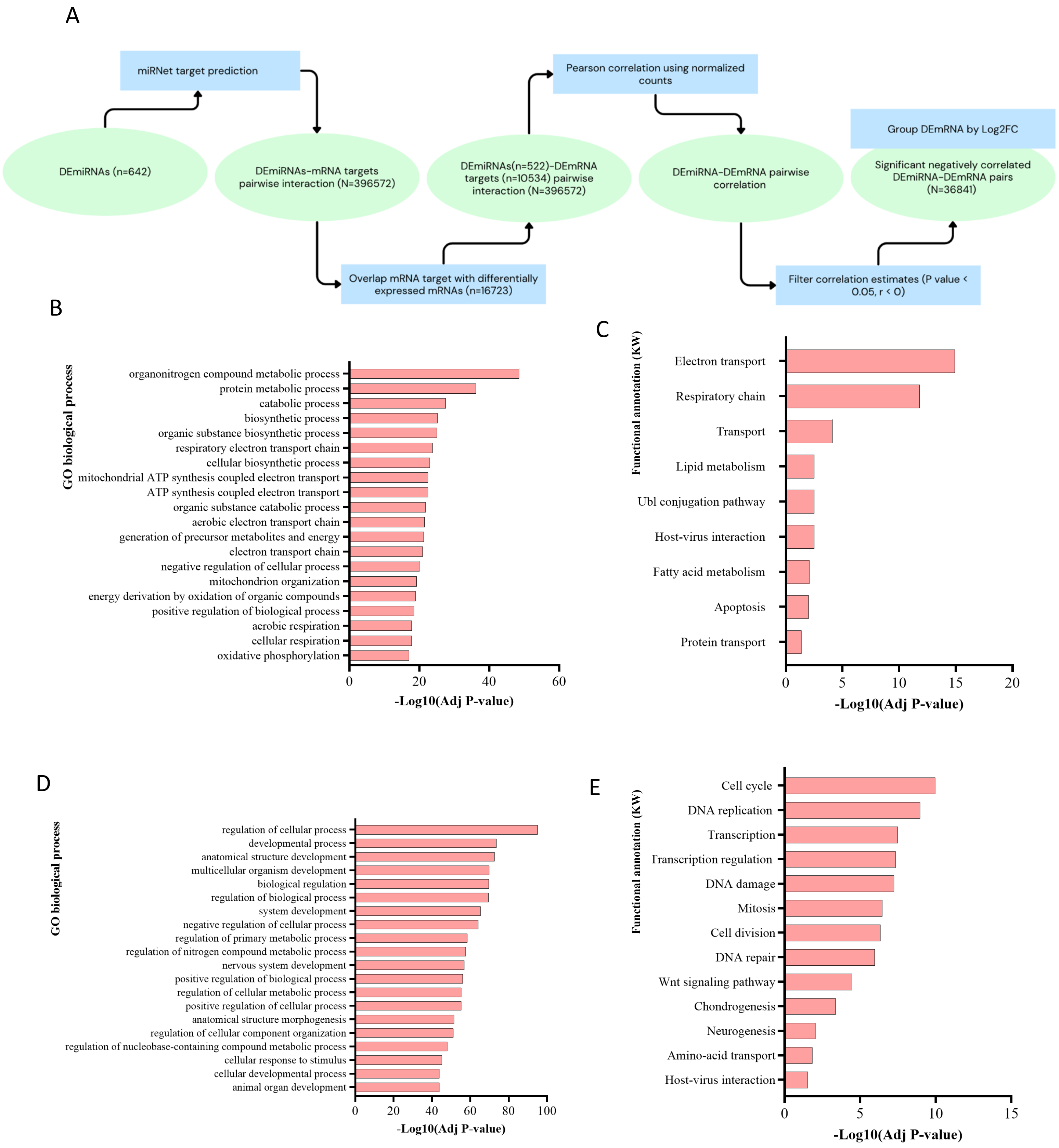

Figure S3. **(A)** Analysis pipeline used to correlate miRNA expression data to mRNA expression data**. (B-E)** Functional enrichment analysis of differentially expressed miRNA-mRNA targets. Gene ontology terms (biological functions) and functional annotation category (uniprot key word biological processes) enriched amongst genes upregulated **(A, B)** and downregulated **(C, D)** in fetal hearts across gestation. Data was analyzed using g:profiler **(A, D)** and DAVID v2023q1 with Benjamini correction **(B, E)**.

A

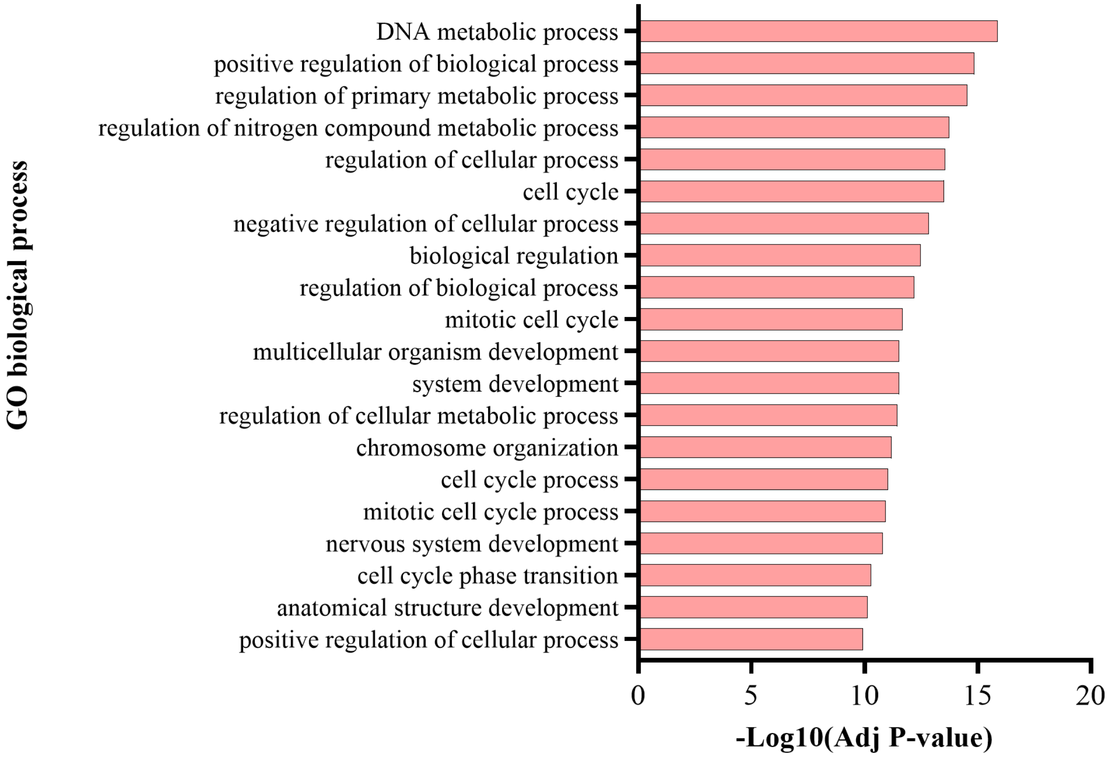

B

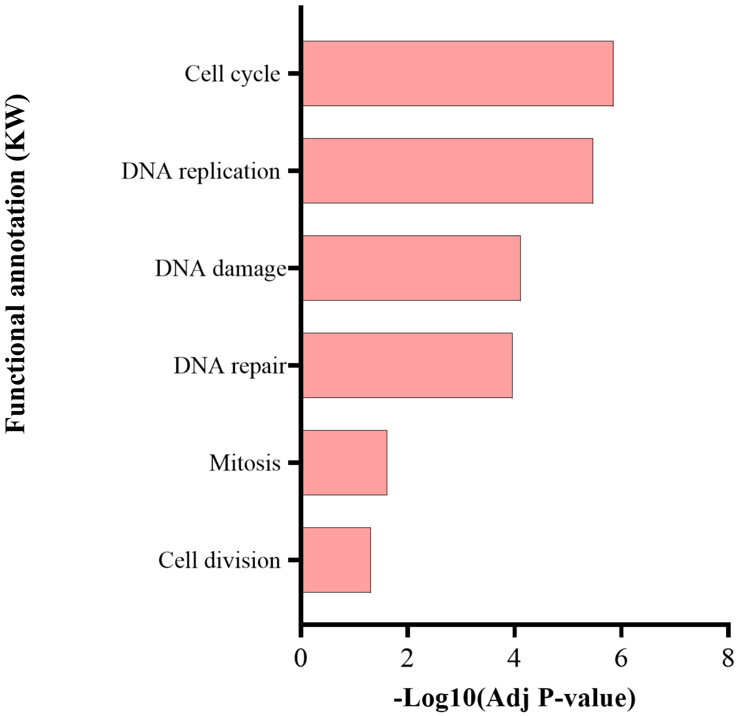

Figure S4. Functional enrichment analysis of top 5% downregulated miRNA-mRNA targets. Gene ontology terms (biological functions) and functional annotation category (uniprot key word biological processes) enriched amongst top 5% downregulated miRNA gene targets in fetal hearts across gestation. Data was analyzed using g:profiler **(A)** and DAVID v2023q1 with Benjamini correction and functional annotation category (KW biological process) **(B)**.

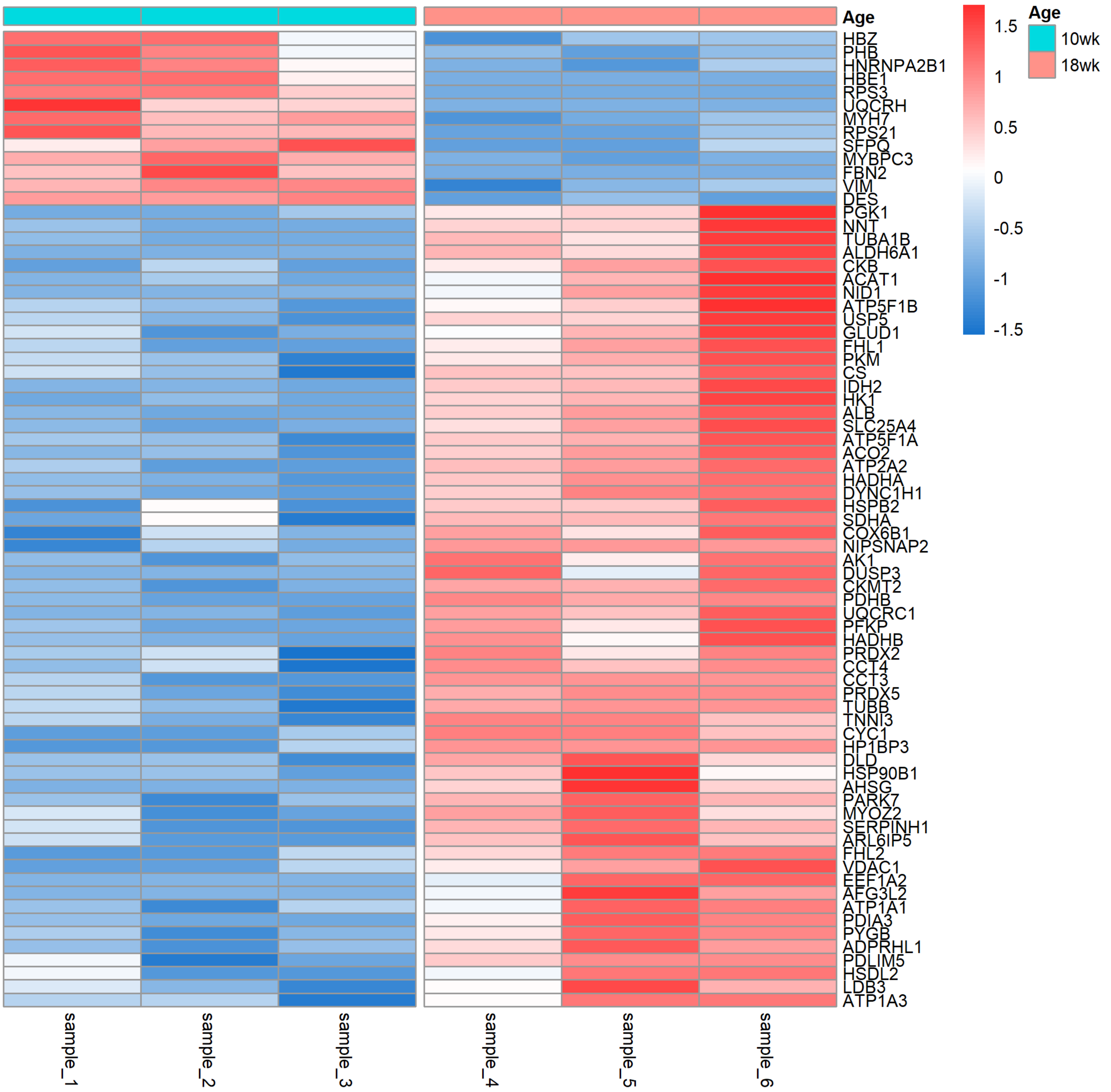

Figure S5. Heatmap displaying normalized abundance (Z-scores) of all (miRNA targets and non-targets) differently abundant proteins between 10 (n=3) and 18-weeks-old (n=3) fetal heart samples (P value < 0.05). Enriched proteins are in red, and depleted proteins are in blue.

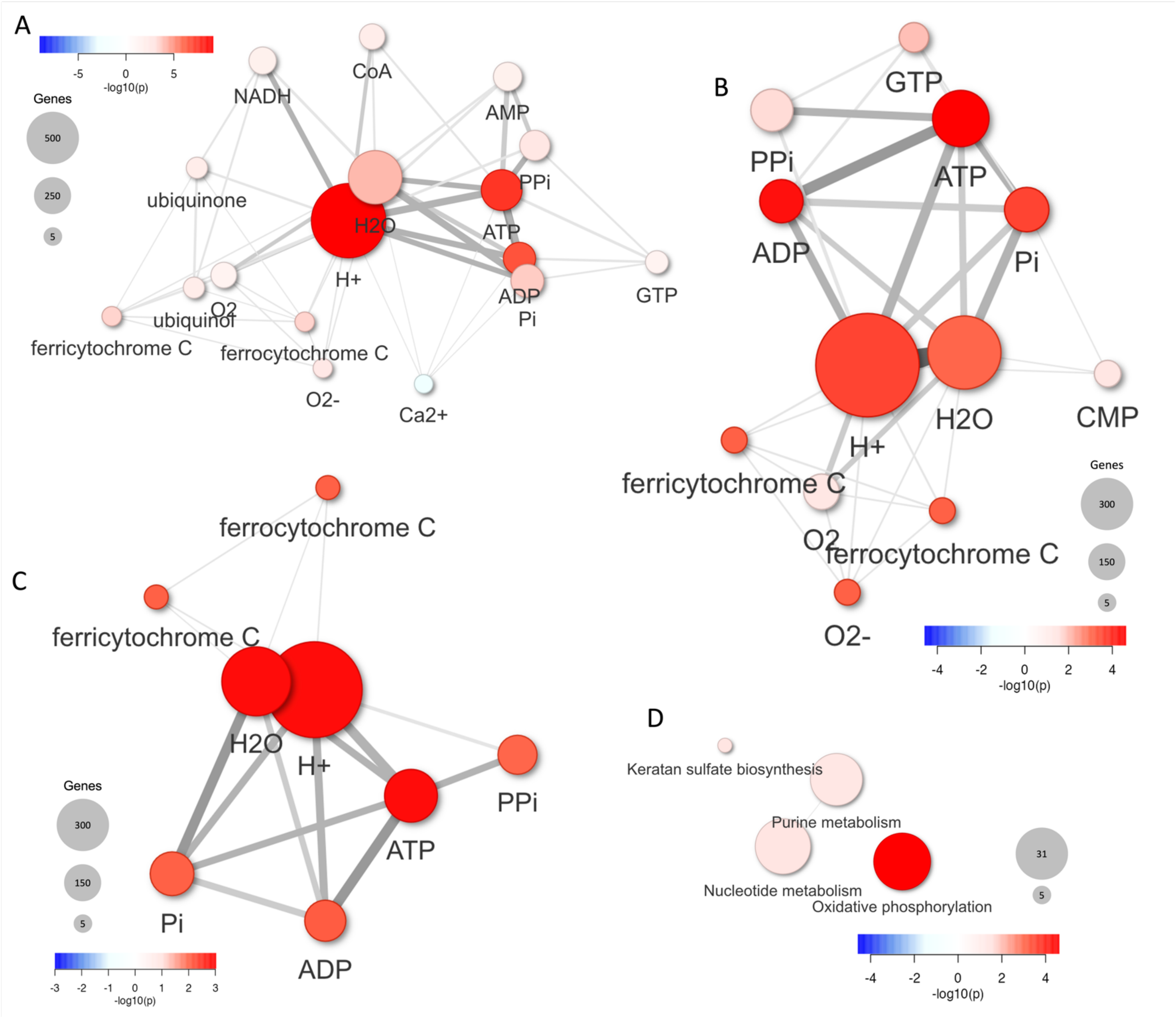

Figure S6. Metabolite reporter analysis of control groups of differentially expressed mRNAs. Network plots of significant metabolites identified in the developing heart using **(A)** 10,533 differentially expressed genes identified as non-targets of miRNAs, **(B)** 6200 randomly selected differentially expressed genes (targets and non-targets) and **(C)** 6200 randomly selected differentially expressed non-target genes. **(D)** Network plot of significant subsystems (metabolic pathways) identified in the developing heart using 6200 randomly selected differentially expressed genes (targets and non-targets). Upregulated metabolites are in red, and downregulated metabolites are in blue. Analysis was performed with piano using distinct-directional adjusted P values with a cut-off of 0.05.
