# Supplementary material for: Integrated small RNA, mRNA and protein omics reveal a miRNA network orchestrating metabolic maturation of the developing human heart
Source: BMC Genomics. 2023 Nov 23;24:709. doi: 10.1186/s12864-023-09801-8 (PMC10668469; doi:10.1186/s12864-023-09801-8)
Supplement: Supplementary file 18 — Additional file 18. Full protocols for protein digestion and identification for mass spectrometry. [file 12864_2023_9801_MOESM18_ESM.doc]

**Additional file 18:** Full protocols for protein digestion and identification for mass spectrometry.

**In-solution tryptic digest**

1. Bring samples up to 100uL total volume (final concentration of 50mM Ammonium Bicarbonate) (150-300ug total protein for lysates)
2. Add 5uL of DTT (200mM DTT in 100mM ammonium bicarbonate), vortex and spin in centrifuge.
3. If sample does not contain Urea, boil for 10min.  Otherwise, leave sample at room temperature for 45min.
4. Add 8uL of iodoacetamide (0.5M iodoacetamide in 100mM ammonium bicarbonate), vortex and spin in centrifuge.
5. Leave sample at room temperature in the dark for 45min.
6. Add 20uL of DTT (200mM DTT in 100mM ammonium bicarbonate), vortex and spin in centrifuge.
7. Leave sample at room temperature for 45min.
8. Add 1ug of Trypsin/50ug protein, gently mix by pipetting and incubate at 37°C overnight.

**OMIX tip cleanup**

**Use only >HPLC grade solvents and water**

1. Adjust sample to 1.0% TFA using 2.5% TFA solution (can use a higher concentration stock of TFA if needed)
2. Wet the tip with 100µl 1:1 ACN:H2O and discard. Repeat.
3. Equilibrate the tip with 100µl 0.1% TFA and discard. Repeat.
4. Aspirate and dispense the sample 3-5 times (up to 10 cycles may be used for improved binding)
5. Aspirate 100uL of 0.1% TFA solution and discard. Repeat
6. Aspirate and dispense 100µl 95% ACN/0.1% formic acid to elute sample
7. Dry sample in speedvac. Dry peptides can be safely stored at -20 for several months until needed.
8. Resuspend peptides in MS buffer A (typically 0.1% formic acid) for analysis, Sonicate if needed.
9. Filter samples through 0.2µ filter prior to loading.

**LC-MS Analysis**

**Chromatography Settings:**

NanoUHPLC: Thermo Scientific Easy nLC 1000

Column: custom packed SilicaTip Emitter (New Objective part# FS360-75-15-N-20)

C18 Resin packed in column: ReproSil-Pur C18-AQ (Dr. Maisch part# r13.aq)

Sample Injection: 5uL

Solvent A: 0.1% formic acid in Water

Solvent B: 0.1% formic acid in Acetonitrile

Flow Rate: 250nL/min

Gradient: 0min-5min, linear gradient to 10%B; 5-93min, linear gradient to 40%B; 93-95min, linear gradient to 95%B; 95-105min, 95%B; 105-106min, linear gradient to 0%B; 106-120min, 0%B (re-equilibriation)

**Mass Spec Settings**

Mass Spectrometer: Thermo Scientific Q-Exactive

Probe: nanoESI

Ionization Mode: Positive

Spray Voltage: 2.5kV

Capillary Temp: 250degC

S-Lens RF Level: 50

Full MS Settings:

Resolution: 70 000

AGC Target: 1e6

Maximum Injection Time: 30ms

Scan Range: 400-2000

Data-Dependent MS2:

Mode: Top 10

Resolution: 17 500

AGC Target:

Maximum Injection Time: 50ms

Isolation window: 0.4 m/z

Scan Range: 200-2000

NCE: 27

Data Processing:

Software: The GPM using X!Tandem search algorithm

Fragment Mass Error: 0.4Da

Parent Mass Error: 20ppm

Ions: a,b,x,y

Database: Human
